# Abnormal brain activity in lumbar disc herniation patients with chronic pain is associated with their clinical symptoms

**DOI:** 10.3389/fnins.2023.1206604

**Published:** 2023-07-28

**Authors:** Cheng Tang, Guangxin Guo, Sitong Fang, Chongjie Yao, Bowen Zhu, Lingjun Kong, Xuanjin Pan, Xinrong Li, Weibin He, Zhiwei Wu, Min Fang

**Affiliations:** ^1^Shuguang Hospital, Shanghai University of Traditional Chinese Medicine, Shanghai, China; ^2^School of Acupuncture-Moxibustion and Tuina, Shanghai University of Traditional Chinese Medicine, Shanghai, China; ^3^Shanghai Municipal Hospital of Traditional Chinese Medicine, Shanghai University of Traditional Chinese Medicine, Shanghai, China; ^4^Department of Neurosurgery, Renmin Hospital of Wuhan University, Wuhan, China; ^5^Research Institute of Tuina, Shanghai Academy of Traditional Chinese Medicine, Shanghai, China; ^6^Yueyang Hospital of Integrated Chinese and Western Medicine, Shanghai University of Traditional Chinese Medicine, Shanghai, China

**Keywords:** lumbar disc herniation, chronic low back pain, resting-state functional magnetic resonance, amplitude of low-frequency fluctuations, anxiety, prefrontal lobe

## Abstract

**Introduction:**

Lumbar disc herniation, a chronic degenerative disease, is one of the major contributors to chronic low back pain and disability. Although many studies have been conducted in the past on brain function in chronic low back pain, most of these studies did not classify chronic low back pain (cLBP) patients according to their etiology. The lack of etiologic classification may lead to inconsistencies between findings, and the correlation between differences in brain activation and clinical symptoms in patients with cLBP was less studied in the past.

**Methods:**

In this study, 36 lumbar disc herniation patients with chronic low back pain (LDHCP) and 36 healthy controls (HCs) were included to study brain activity abnormalities in LDHCP. Visual analogue scale (VAS), oswestry disability index (ODI), self-rating anxiety scale (SAS), self-rating depression scale (SDS) were used to assess clinical symptoms.

**Results:**

The results showed that LDHCP patients exhibited abnormally increased and diminished activation of brain regions compared to HCs. Correlation analysis showed that the amplitude of low frequency fluctuations (ALFF) in the left middle frontal gyrus is negatively correlated with SAS and VAS, while the right superior temporal gyrus is positively correlated with SAS and VAS, the dorsolateral left superior frontal gyrus and the right middle frontal gyrus are negatively correlated with VAS and SAS, respectively.

**Conclusion:**

LDHCP patients have brain regions with abnormally increased and abnormally decreased activation compared to healthy controls. Furthermore, some of the abnormally activated brain regions were correlated with clinical pain or emotional symptoms.

## Introduction

Chronic low back pain (cLBP), as a common clinical disease, is one of the leading causes of disability (Wang et al., 2022) and remains a major medical and social problem worldwide. The 2019 Global Burden of Disease Study pointed out that approximately 568.4 million people suffer from cLBP (Chen et al., 2022). One of the major factors causing chronic low back pain is lumbar disc herniation (LDH). As clinical neuropathic pain, LDH is mainly due to intervertebral disc injuries and degenerative changes with age. Rupture of the fibrous ring and protrusion of the nucleus pulposus tissue causes physical compression of the paravertebral nerve roots, which results in low back pain and dysfunction (Martin et al., 2002; Wang et al., 2021). On the other hand, the immune system will recognize the exposed nucleus pulposus and produce multiple proinflammatory factors, including interleukin-1, prostaglandin E2, 5-hydroxytryptamine, and tumor necrosis factor, to increase the sensitivity and extent of pain (Zhao et al., 2019). Studies have found that negative emotions, such as anxiety and depression, can exacerbate pain, with the increasing severity and duration of chronic pain (Sheng and Zhang, 2019; Li et al., 2021; Kao et al., 2022). A study on the chronic pain model of LDH also pointed out that LDH is often accompanied by depression, especially in females with severe pain and a longer course of the disease (Cai et al., 2019). As one of the main specificity factors of cLBP, LDH is frequently lack of compatibility between the severity of lumbar spine CT/MRI findings and clinical symptoms in clinical practice. For example, some patients have a large herniated nucleus pulposus but no significant clinical symptoms, while others have unbearable pain, weakness, and other clinical symptoms with only a bulging lumbar disc. We speculate that the emotional state of the patient regarding LDHCP may be an important factor in this phenomenon. Emotional experiences and psychological states can influence clinical pain symptoms through functional and structural changes in the central nervous system and should therefore also be taken into account in the diagnosis and treatment of LDH (Mu et al., 2019; Price and Duman, 2020).

As scientific exploration of the brain continues to extend, more and more imaging techniques are making it possible to accurately assess pain and emotional interactions. The amplitude of low frequency fluctuation (ALFF), regional homogeneity, and functional connectivity are commonly used to assess pathological changes in functional magnetic resonance imaging (fMRI) studies. These neuroimaging methods can quantify and visualize higher central changes in cLBP (Weizman et al., 2018; Huang et al., 2020; Li et al., 2020). However, previous fMRI studies have mostly failed to classify cLBP specifically or nonspecifically according to etiology, which may make the findings somewhat controversial. For example, Zhang et al. reported that cLBP patients’ ALFF is increased in the post−/precentral gyrus, paracentral lobule (PCL)/supplementary motor area (SMA), and PCL/SMA ALFF reliably discriminated cLBP patients from HCs in an independent cohort (Zhang et al., 2019). Another team argued that cLBP patients had reduced ALFF in the right posterior cingulate cortex/precuneus cortex and left primary somatosensory cortex (S1), but elevated ALFF in the right medial prefrontal cortex, right middle temporal gyrus, bilateral inferior temporal gyrus, bilateral insula, and left cerebellum (Zhang et al., 2017). Thus, it is important to classify whether chronic low back pain is a specific etiology or not. It has been reported that cerebellar associated with injury perception and endogenous pain modulation, inhibitory cerebellar t-DCS would increase pain perception and reduced endogenous pain inhibition while excitatory cerebellar t-DCS increased endogenous pain inhibition (Stacheneder et al., 2022). Similarly, we can try to find the specific brain regions with altered brain function in LDHCP and conduct interventional longitudinal studies on the corresponding brain regions in subsequent studies. Studying its pain-causing brain function pathological features by fMRI analysis methods would help LDH clinical diagnosis and treatment, but regretfully there are few corresponding studies. Among fMRI analysis methods, ALFF can directly reflect the magnitude of baseline changes in the brain blood oxygen level-dependent effect (BOLD) signal and indirectly indicate the intensity of local neuronal spontaneous activity in the brain. ALFF is considered to be one of the most common methods for observing changes in brain function at rest and is widely used in brain function studies of pain-related diseases (Zang, 2016; Du et al., 2018; Pan et al., 2018; Ge et al., 2022). Therefore, we used a data-driven ALFF analysis to explore differences in brain activity between patients with lumbar disc herniation chronic low back pain (LDHCP) and healthy controls (HCs). The Visual Analog Scale (VAS), Oswestry Disability Index (ODI), Self-Rated Anxiety Scale (SAS), and Self-Rated Depression Scale (SDS) were used to assess clinical symptoms and explore their association with abnormal brain regions in LDHCP. We hypothesized that LDHCP will result in abnormal brain activity and the abnormal brain activity in LDHCP would be related to their clinical symptoms.

## Materials and methods

### Subjects

36 LDHCP patients were recruited at Yueyang Hospital of Integrated Traditonal Chinese and Western Medicine, Shanghai University of Traditonal Chinese Medicine (Shanghai, China) from December 2021 to December 2022. The clinical trial was registered on November 24, 2021 at the China Clinical Trials Registry with registration number ChiCTR2100053542. 36 age-and sex-matched HCs were recruited from communities. All subjects underwent Mini-mental State Examination (MMSE) test prior to enrollment to ensure the subjects were cognitively normal.

The inclusive criteria of LDHCP were as follows: (1) Age between 18 and 65 years, right-handed; (2) CT or MRI shows herniated disc in the lumbar spine and suffering from low back pain for at least 3 months or longer; (3) VAS score ≥ 30/100 points; (4) ODI score ≥ 20/100 points; and (5) not receiving pain therapy for at least 1 month before our enrollment. The inclusive criteria of HCs were as follows: (1) aged between 20 and 65 years; (2) right-handed; (3) no LDH history and related symptoms; and (4) without negative emotions.

The exclusive criteria were used for both HC and LDHCP groups: (1) subjects with organic brain lesions or history of brain surgery; (2) subjects with contraindications to MRI; (3) pregnant or lactating subjects; (4) subjects with alcohol or drug dependence; (5) subjects with other serious co-morbidities; and (6) subjects with an MMSE score less of than 27 points (Folstein et al., 1975).

### Clinical assessment

This study used VAS, ODI, SAS, SDS to assess LDHCP’s somatic pain, functional activity and related anxiety and depression status. VAS is reliable in assessing the severity of low back pain and in predicting disability (Thong et al., 2018; Shafshak and Elnemr, 2021). VAS divides the pain level evenly on a straight line with 10 scales into two endpoints: no pain and extreme pain, corresponding to scores of 0 and 10, respectively. ODI is a effective and validated scale for measuring disability in patients with low back pain and has high-quality psychometric properties in terms of construct validity, test–retest reliability and internal consistency (Chapman et al., 2011; Sheahan et al., 2015; Arpinar et al., 2020). It consists of 10 scoring items, namely back pain and leg pain, personal care, lifting heavy objects, walking, sitting, standing, sleeping, sexual life, social life, and traveling. The patient’s performance in each item is scored on a scale of 6 degrees from mild to severe, corresponding to grades 0 to 5 points. The SAS is very similar to the SDS and is a fairly simple clinical tool for analyzing patients’ subjective symptoms (Thurber et al., 2002; Dunstan and Scott, 2020). It is suitable for adults with symptoms of anxiety or depression and has a wide range of applications. SAS has high reliability estimates while SDS has good sensitivity and specificity (Knight et al., 1983; Turner and Romano, 1984). It is important to emphasize that SAS and SDS tests are performed within 1 h prior to each MRI to quantify the subject’s state of mind as much as possible.

### Magnetic resonance imaging data acquisition

Magnetic resonance imaging was performed by a 3.0T SIEMENS MAGNETOM (Germany) with a 32 channel head coil at Yueyang Hospital of Integrated Traditional Chinese and Western Medicine Affiliated to Shanghai University of Traditional Chinese Medicine, China. All subjects wore cotton earplugs to reduce noise interference and their heads were fixed with a soft foam pad to reduce head movement bias. During the MRI scan, all subjects were asked to remain awake and relaxed, with no excessive head movements or mental activity allowed, otherwise the data collected would not be included in the study. The scanning parameters were set as follows: (1) functional MRI: 33 interleaved axial slices, matrix size = 64 × 64, field of view (FOV) = 220 mm × 220 mm, repetition time (TR) = 2,000 ms, echo time (TE) = 30 ms, flip angle = 90 degrees, slice thickness = 4 mm, gap = 0 (voxel size3.4 × 3.4 × 4.0), number of volumes = 240. (2) structural MRI: Sequence = SPGR, sagittal slices, slice number = 160, matrix size = 256 × 256, FOV = 256 × 256 mm, TR/TE = 1900/2.93 ms, flip angle = 9 degrees, slice thickness = 1, gap = 0 (voxel size = 1 × 1 × 1). After the scan, the subject was asked if he/she had fallen asleep during the scan and if he/she gave an accurate or vague answer, the subject’s MRI data was also excluded. All scans were performed by the same MRI physician who had been formally trained by Siemens.

### Data preprocessing

RESTplus (Resting-state fMRI data analysis Toolkit),^1^ a brain imaging data processing and analysis software based on statistical parametric mapping (SPM12),^2^ was used for rs-fMRI data preprocessing. The procedure was as follows: (1) convert the original Dicom files to NIFTI format; (2) remove the first 10 time points to stabilize the longitudinal magnetization; (3) slice timing to eliminate differences in acquisition times between adjacent scan levels; (4) realign to calibrate the subject’s head position at different time points in the scan and remove data from patients with head motion >3 mm and rotation >3° in any direction; (5) normalizing to Montreal Neurological Institute (MNI) space by Diffeomorphic Anatomical Registration Through Exponentiated Lie Algebra (DARTEL) using T1 image new segment; (6) smoothing of the functional image aligned to the MNI standard space using a 6-mm full width at half maximum (FWHM) kernel; (7) detrending to reduce thermal interference from the MR coil and noise generated by the subject’s personal factors (e.g., breathing, heartbeat, etc.); and (8) low-frequency filtering (Filter): 0.01–0.08 Hz signal is selected to filter the image for calculation to eliminate interference from other high-frequency signals.

### Amplitude of low-frequency fluctuations calculation

ALFF values were calculated using RESTplus (Resting-state fMRI data analysis Toolkit, see text footnote 1). The procedure for calculating the ALFF for each voxel in the brain is as follows: (1) pass the time series of each voxel through a 0.01–0.08 Hz band-pass filter after removing the linear drift; (2) obtain the power spectrum by performing a fast Fourier change on the filter results; (3) square the power spectrum; (4) calculate the average of the power spectrum within 0.01–0.08 Hz as the ALFF; and (5) divide the ALFF divided by the average ALFF of all voxels in the whole brain to obtain the normalized ALFF (mALFF).

### Statistical analysis

SPSS 24 (IBM, United States) was used for the statistical analysis of demographic and clinical data in this study. For the count data, frequency distributions were described and statistical differences between groups were analyzed using the chi-square test; for the measurement data, the *t*-test was used if the data conformed to a normal distribution, and if not, the Mann–Whitney *U* test for independent samples was used. Statistical tests were all performed using a two-tailed test, *α* = 0.05, and differences were considered statistically significant if *p* < 0.05. Imaging data statistics were analyzed using SPM software. Voxel-by-voxel statistics were performed using a general linear model (GLM) with two-sample *t*-tests for subjects in both groups, with gender and age as covariates. Family-wise error (FWE) was used to correct the results for multiple comparisons, with a voxel-level significance threshold of *p* < 0.001 and a cluster-level significance threshold of *p* < 0.05. If the continuous variables conform to normality, Pearson correlation coefficient will be used to assess whether there is a correlation between signal values of brain region activity and clinical symptom scores. If the continuous variables do not conform to normality, correlations between indicators are calculated using the Spearman correlation method. *P* < 0.05 is the threshold of statistical difference for correlation analysis.

## Results

### Demographic and clinical data

A total of 72 participants were selected for this study, including 36 (12 male/24 female) LDHCP patients and 36 (14 male/22 female) HCs. Table 1 shows the demographic and clinical characteristics of the participants. As shown in Table 1, there were no significant differences in gender (*p* = 0.624), age (*p* = 0.456), weight (*p* = 0.061), occupation (*p* = 0.551), MMSE (*p* = 0.11) between LDHCP and HCs. Subjects in both groups were comparable.

**Table 1:** Demographic characteristics of the LDH and HC groups.

	LDHCP	HCs	*p*
Gender	12/24	14/22	0.624^a^
Age	38.58 ± 1.93	38.17 ± 2.48	0.456^b^
Weight (KG)	61 (55, 70)	69 (59, 74)	0.061^c^
Occupation	8/28	6/30	0.551^a^
(Physically / Non-physically)			
MMSE	27.02 ± 1.20	27.47 ± 0.99	0.11^b^
VAS	6 (5, 7)	/	NA
ODI	19 (14, 27)	/	NA
SAS	41.30 ± 0.92	/	NA
SDS	41.19 ± 1.39	/	NA

^a^χ^2^-test; ^b^Two sample t-test; ^c^Non-parametric-tests.

LDHCP, Lumbar disc herniation patients with chronic pain; HCs, health controls; MMSE, Mini-mental State Examination; VAS, Visual Analog Scale; ODI, Oswestry Disability Index Questionnaire; SAS, self-rating anxiety scale; SDS, Self-rating depression scale.

### Amplitude of low-frequency fluctuations analysis

**Figure 1:**
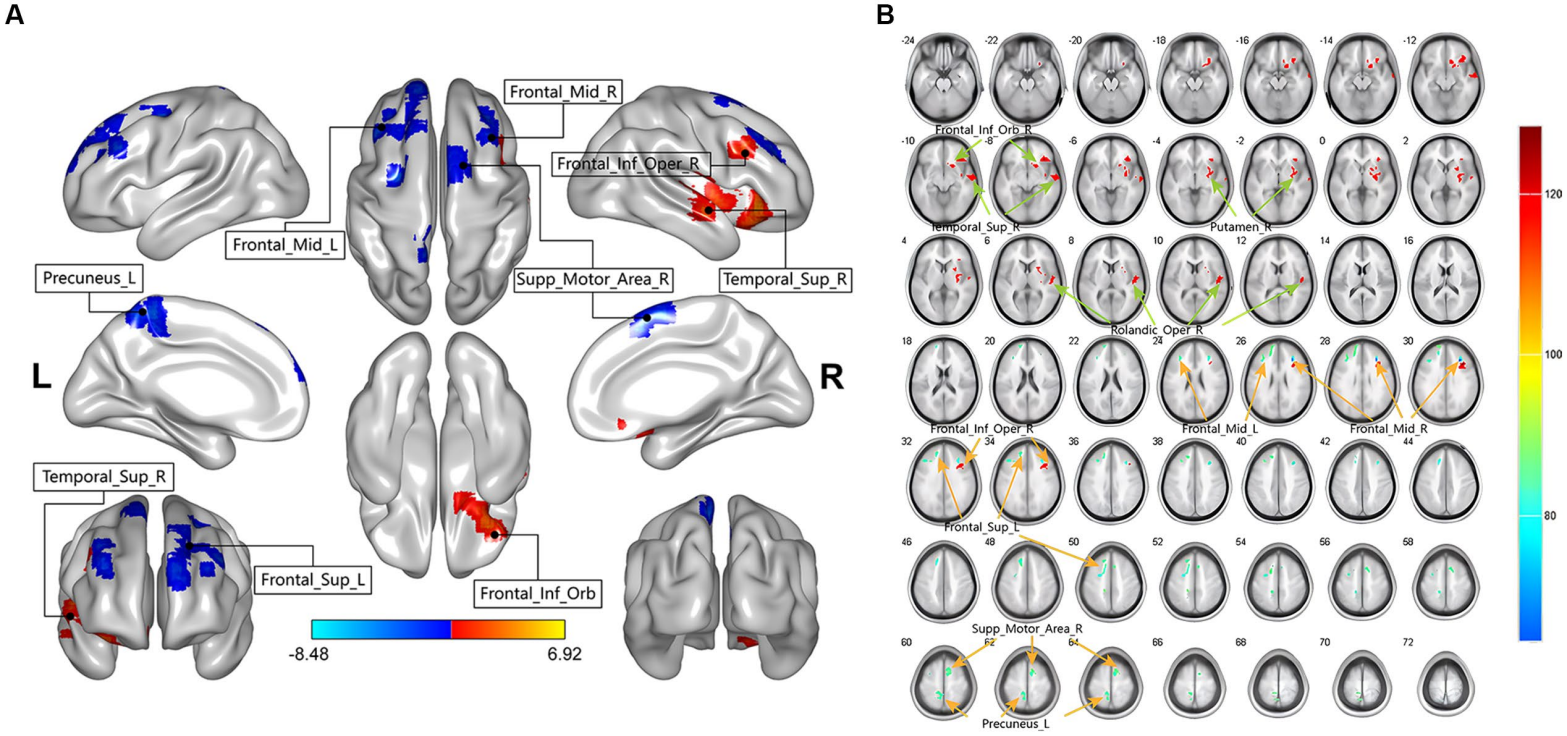
The two-sample t-test showing significant differences in mean-standardized amplitude of low-frequency fluctuations (mALFF) between LDHCP patients and HCs. The color bar indicates T-values. **(A)** Spatial location map of abnormally activated brain regions in LDHCP patients; **(B)** Layers section of abnormally activated brain regions in LDHCP patients.

In this study, there were differential brain areas with increased and decreased ALFF values in the LDHCP patients compared to the HC group (Figure 1A). Brain areas with increased ALFF were mainly located in Right inferior frontal gyrus, orbital part (Frontal_Inf_Orb_R); Right superior temporal gyrus (Temporal_Sup_R); Right lenticular nucleus, putamen (Putamen_R); Right rolandic operculum (Rolandic_Oper_R); Right Inferior frontal gyrus, opercular part (Frontal_Inf_Oper_R); (Table 2 and Figure 1B The brain area pointed by the green arrow); brain areas with decreased ALFF were mainly located in Left Superior frontal gyrus (Frontal_Sup_L), Left middle frontal gyrus (Frontal_Mid_L), Right middle frontal gyrus (Frontal_Mid_R), Left Precuneus (Precuneus_L), Right Supplementary motor area (Supp_Motor_Area_R) (Table 2 and Figure 1B The brain area pointed by the yellow arrow).

**Table 2:** Brain region with a significant difference in mALFF between two groups.

Brain regions	Cluster size	Peak MNI coordinates			*t*-values	*P*
AAL	(voxels)	*x*	*y*	*z*		
LDHCP > HC						
Frontal_Inf_Orb_R	124	36	30	−9	5.9883	<0.001
Temporal_Sup_R	53	66	−9	−9	5.2409	0.017
Putamen_R	86	27	−9	0	4.9789	0.001
Rolandic_Oper_R	41	51	−15	9	5.2708	0.049
Frontal_Inf_Oper_R	44	36	21	30	6.9224	0.038
LDHCP < HC						
Frontal_Sup_L	114	−15	27	45	−5.3203	<0.001
Frontal_Mid_L	45	−36	27	30	−5.3483	0.034
Frontal_Mid_R	51	30	30	27	−8.4784	0.02
Precuneus_L	70	−9	−39	63	−4.8403	0.004
Supp_Motor_Area_R	56	9	3	63	−5.0943	0.013

Results were corrected for multiple comparisons using the Family Wise Error (FWE) with a cluster level significance threshold of *P* < 0.05. Frontal_Inf_Orb_R, Right inferior frontal gyrus, orbital part; Temporal_Sup_R, Right superior temporal gyrus; Putamen_R, Right lenticular nucleus; Rolandic_Oper_R, Right rolandic operculum; Frontal_Inf_Oper_R, Right Inferior frontal gyrus, opercular part; Frontal_Sup_L, Left Superior frontal gyrus; Frontal_Mid_L, Left middle frontal gyrus; Frontal_Mid_R, Right middle frontal gyrus; Precuneus_L, Left Precuneus; Supp_Motor_Area_R, Right Supplementary motor area.

### Clinical symptoms correlation

**Figure 2:**
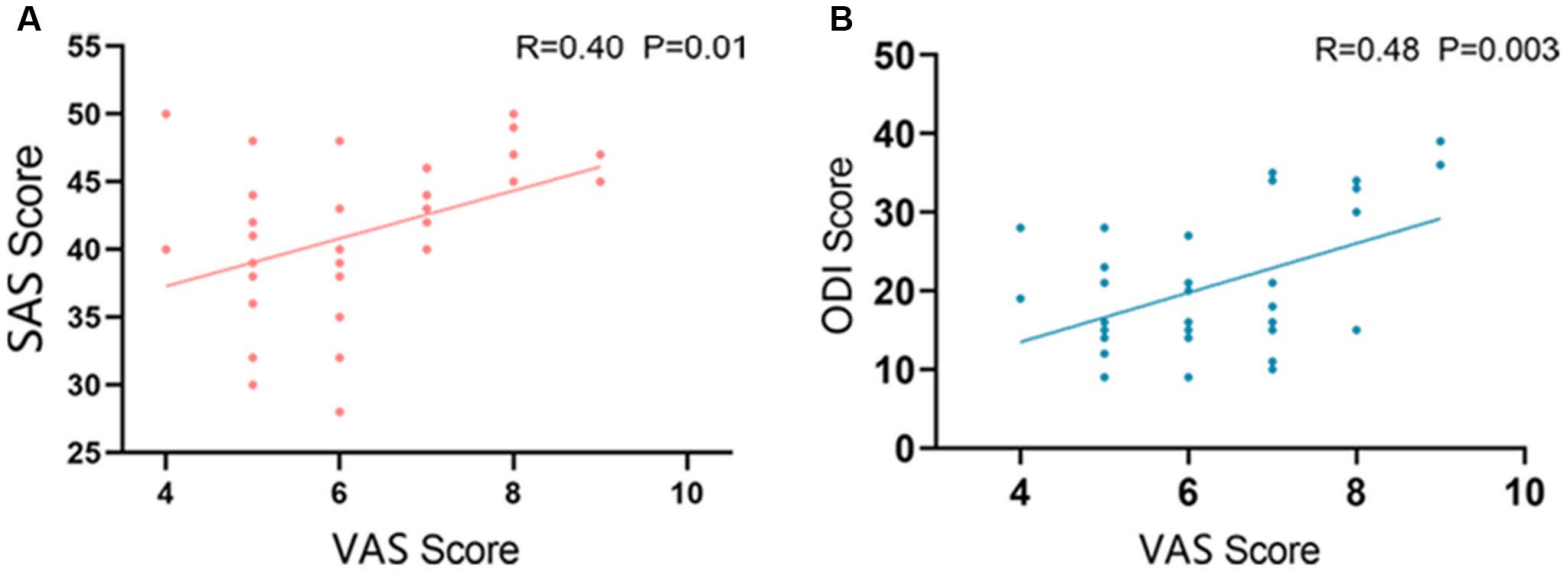
Correlation between clinical symptoms. **(A)** The correlation between VAS and ODI (R=0.47 *P*=0.034); **(B)** The correlation between VAS and SAS (R=0.40 *P*=0.014).

In LDHCP patients’ clinical symptom scores, the VAS scores showed a significant Pearson correlation with the ODI scores and the SAS scores, respectively. The correlation coefficient between VAS and ODI is 0.47, *p* = 0.034 (Figure 2A) and between VAS and SAS scores is 0.40, *p* = 0.014 (Figure 2B).

### Clinical-magnetic resonance imaging correlations

**Figure 3:**
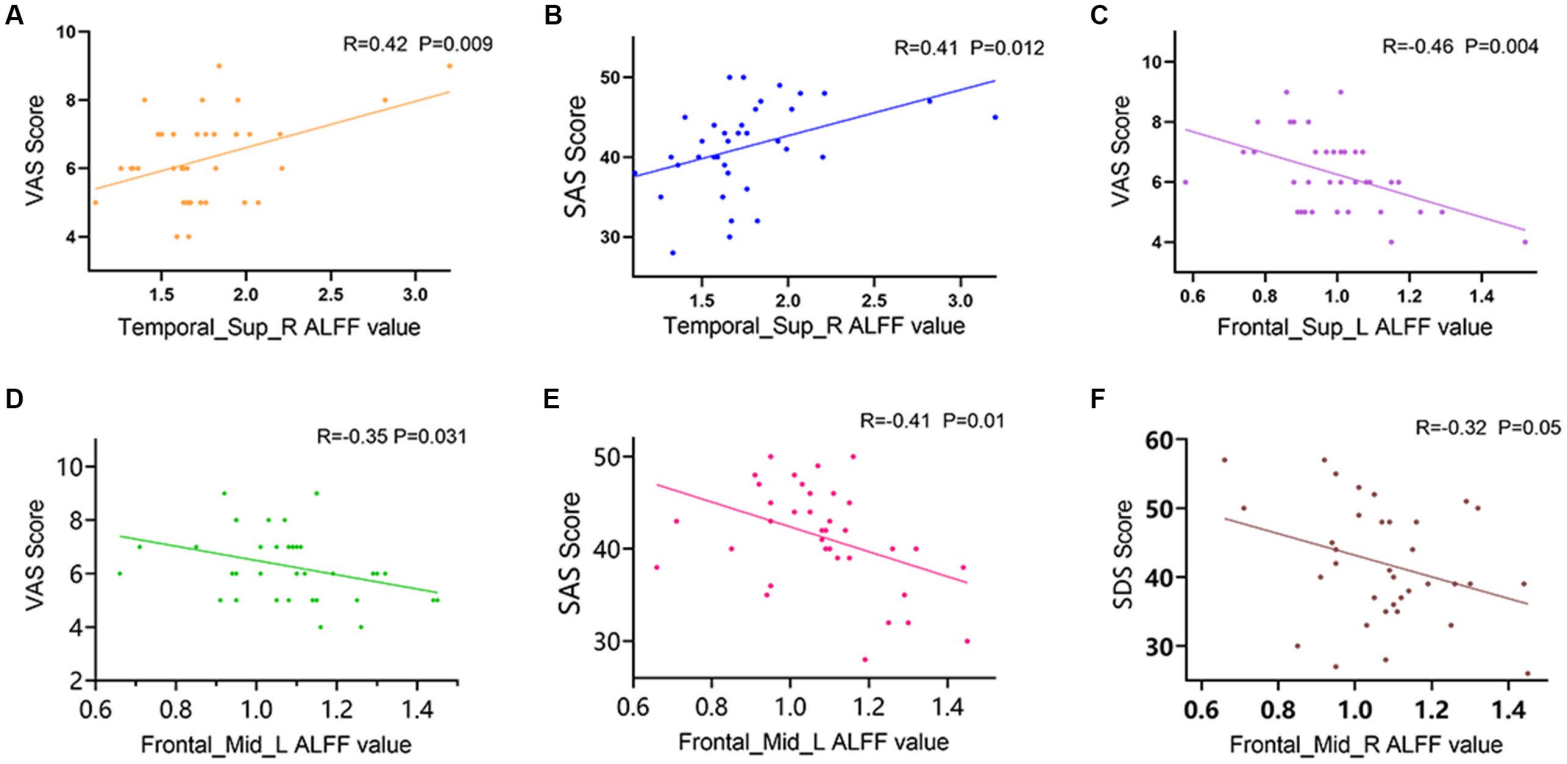
Correlation between mALFF values of abnormally activated brain regions and clinical symptoms in LDHCP patients. **(A)** Positive correlation between the mALFF values in the Temporal_Sup_R and the VAS scores; **(B)** Positive correlation between the mALFF values in the Temporal_Sup_R and the SAS scores; **(C)** Negative correlation between the mALFF values in the Frontal_Sup_L and the VAS scores; **(D)** Negative correlation between the mALFF values in the Frontal_Mid_L and the VAS scores; **(E)** Negative correlation between the mALFF values in the Frontal_Mid_L and the SAS scores; **(F)** Negative correlation between the mALFF values in the Frontal_Mid_R and the SDS scores.

In LDHCP patients, Right superior temporal gyrus (Temporal_Sup_R) is positively correlated with VAS scores (*R* = 0.42, *p* = 0.009) (Figure 3A); Right superior temporal gyrus (Temporal_Sup_R) is positively correlated with SAS (*R* = 0.41, *p* = 0.012) (Figure 3B); Left Superior frontal gyrus (Frontal_Sup_L) is negatively correlated with VAS scores (*R* = −0.46, *p* = 0.004) (Figure 3C); Left middle frontal gyrus (Frontal_Mid_L) is negatively correlated with VAS scores (*R* = −0.35, *p* = 0.031) (Figure 3D); Left middle frontal gyrus (Frontal_Mid_L) is negatively correlated with SAS scores (*R* = −0.41, *p* = 0.01) (Figure 3E); Right middle frontal gyrus (Frontal_Mid_R) is negatively correlated with SDS (*R* = −0.32, *p* = 0.05) (Figure 3F).

## Discussion

This study is based on the 0.01–0.08 Hz classic frequency band in ALFF analysis to explore the brain regions where local neural activity changes in LDHCP patients compared with HC group and the possible links between these changes and clinical symptoms. In our study, we found that LDHCP patients have functional abnormalities in several brain regions in the resting state. Among them, the right superior temporal gyrus, the left dorsolateral superior frontal gyrus, and the left and right middle frontal gyrus were correlated with clinical pain or mood-related scores, respectively. Correlation analysis also found a significant correlation between VAS and ODI, but no correlation was found between brain regions and ODI. The absolute value of the correlation coefficient R ranged from 0.32 to 0.46, which was considered a low correlation in a mathematical sense. Several studies have pointed out that patients with chronic pain tend to have negative emotions. In turn, negative emotions such as anxiety, depression, fear and catastrophic beliefs contribute to the pain perception and disability of patients with chronic low back pain, affecting their life quality and functional status (Bletzer et al., 2017; Le Borgne et al., 2017; Serbic and Pincus, 2017; Koechlin et al., 2018). It is in accordance with our results of correlation between clinical symptoms.

In our study, the right superior temporal gyrus, left dorsolateral superior frontal gyrus, and left middle frontal gyrus of LDHCP patients are associated with VAS, while the right superior temporal gyrus and middle frontal gyrus are associated with anxiety or depression. The frontal lobe, as an emotion regulation center, is related to attention, working memory and verbal behavior, and can receive rich emotional information, which is closely related to anxiety. Peng et al. (2019) stated that patients with anxiety depression have significantly reduced gray matter volumes in the right inferior frontal gyrus and orbitofrontal gyrus compared to non-anxious depressed and healthy controls. Patients with generalized anxiety disorder have been reported a reduced network connectivity in the prefrontal lobes (Wang W. et al., 2016), while major depressive disorder with somatic symptoms also shows lower ReHo values in the right middle frontal gyrus compared to HC (Geng et al., 2019). A meta-analysis of magnetic resonance spectra also proved that anxiety is associated with metabolic dysfunction in several brain regions, including the dorsolateral prefrontal and hippocampus (Delvecchio et al., 2017). These previous studies have explored the possibility that abnormal activation of frontal subregions may be a potential target for the development of anxiety and depression from a variety of perspectives, including structural, local functional activity, whole brain network connectivity and alterations in metabolic transmitters. In addition to the frontal regions of the brain, the temporal lobe has also been linked to anxiety. The temporal lobe serves as an important node involved in the top-down process of anxiety emotion regulation in the frontal-amygdala loop (Montag et al., 2013). A graph theory study based on the topological properties of brain networks also found that the clustering coefficients of the inferior temporal gyrus were significantly higher in patients with anxiety disorders than in non-anxiety disorders, suggesting that abnormalities in temporal lobe function are associated with the neural network mechanisms by which anxiety disorders occur (Fang et al., 2017).

It is well known that the various parts of our brain do not function independently of each other. Spatially distributed brain areas interact with each other through local information and connections within and between networks to perform different functions. The prefrontal cortex (PFC) abnormalities not only affect negative emotions, but also exhibit a complex association with pain. The PFC, as the higher center of nociceptive encoding, is able to integrate nociceptive sensory and emotional information to produce memory, cognition and evaluation of pain, relying on its connections with brain regions such as the hippocampus, periaqueductal gray matter of the midbrain, thalamus and amygdala (Wang et al., 2020). When dealing with stimuli from acute and chronic pain, the PFC undergoes changes in neurotransmitters, gene expression, glial cells and neuroinflammation, which cause changes in its structure, activity and connectivity (Davey et al., 2019). The gray matter volume of the mPFC extending to the ACC region was found to be significantly reduced in patients with CLBP (Yuan et al., 2017), and the functional connectivity of the mPFC/ACC with other regions in the DMN was reduced (James et al., 2001; Tu et al., 2019). The middle frontal gyrus, as a central region in the prefrontal cortex for processes related to cognitive control and emotion regulation, is more sensitive to pain perception and sensation. Wang J. J. et al. (2016) reported reduced ALFF values in the orbitofrontal cortex and right middle frontal gyrus bilaterally in migraine patients compared to HCs and were associated with depressive co-morbidity. In addition, dorsolateral prefrontal cortex (DLPFC), one of the main components of the central control network ECN, are not only involved in higher cognitive functions but also have important responsibilities in the nociceptive downstream inhibitory pathway, playing a facilitative or inhibitory role in pain (Beltran Serrano et al., 2019). Several studies have pointed out that cLBP patients have significantly reduced gray matter volume in the frontal middle gyrus or DLPFC, as well as reduced functional connectivity of the left lateral prefrontal lobes in the disabled subgroup of cLBP patients compared to the non-disabled subgroup, suggesting that our pain chronicity may be related to abnormalities in the downstream inhibitory function of the DLPFC. Pain not only is a physical phenomenon, but also an emotional experience. There are relatively few studies relating the temporal lobe to pain, but the temporal lobe is implicated in emotion regulation and memory processing and may be involved in pain-related emotional processing and memory formation (Houde et al., 2020). Peng suggested that somatic pain VAS scores in the Parkinson’s with pain group were associated with activation of the left middle temporal gyrus, a brain region associated with nociception, by a mechanism that may be due to a dopamine deficiency associated with mood disorders that enhances the propagation of injury signals and pain sensitivity (Peng, 2020).

Interestingly, in the correlation results of this study, we found a positive correlation between pain and anxiety, while the right superior temporal gyrus and the left middle frontal gyrus correlated with both clinical pain and anxiety scores. This suggests to us that abnormalities in the right superior temporal gyrus and left middle frontal gyrus may be the main and mediating factor for the occurrence of pain and emotion interaction in LDHCP patients. Future analysis and validation of large sample brain imaging cohort studies could focus on these 2 brain regions as areas of interest. After a large sample or multicenter validation, an attempt could also be made to use changes in these two brain regions as an evaluation indicator for clinical interventions. By observing the changes in brain region activities before and after different interventions, the efficacy of different treatments for LDHCP may thus be evaluated. Other brain areas differing in LDHCP patients compared to the HC group in this study, which did not show a correlation with clinical scale scores, also have an important influence in pain and emotional processing. Studies have shown that the DMN is one of the main networks affected by chronic pain (Jones et al., 2020), being modulated and reorganized by chronic pain. The precuneus, a functional center of the default mode network which modulates pain sensitivity and pain thresholds, is structurally and functionally altered in chronic pain (Zhang et al., 2014; Wang et al., 2019). The right inferior orbital frontal gyrus is anatomically connected to the limbic system and other prefrontal brain regions and is a superior integration center for emotional processing. Patients with depression showed reduced clustering coefficients in the inferior orbital frontal gyrus and reduced hemodynamic activation (Zhang et al., 2020; Feng et al., 2021).

However, some LDHCP studies have shown results different from our findings. Wen et al. found a completely different finding from ours, they point out that the LDHCP patients exhibited increased fALFF in right lingual gyri in the conventional band, and showed increased fALFF in left Cerebelum_Crus1 in the slow-4 band (Wen et al., 2022). In addition to finding similar results to our study in the prefrontal cortex or temporal lobe, Zhou et al. (2018) also noted that LDHCP patients had abnormal activation in brain regions such as the insula, cingulate gyrus, posterior cerebellum, inferior parietal lobule, middle occipital gyrus, and postcentral gyrus. It is worth noting that patients recruited by Zhou et al. (2018) had pain in their legs in addition to cLBP. We consider these controversial findings mainly for the following three reasons. First, different pain locations and differences in the distribution of subjects in terms of age, gender, and disease duration may be the main reasons for the different study results (von Leupoldt et al., 2011; Zhao et al., 2013; Wink, 2019; Tsvetanov et al., 2021). Second, different brain imaging data acquisition machines, processing software, and preprocessing steps used by different study groups may make differences in the study results (Murphy et al., 2007; Ashburner, 2009; Goto et al., 2013; Qing et al., 2015; Shirer et al., 2015; Hartwig et al., 2017; Gargouri et al., 2018). Third, the correction methods and thresholds set by different teams during the statistical analysis may make differences in the study results (Durnez et al., 2014; Fasiello et al., 2022; Noble et al., 2022). In the future, the academic community should endeavor to establish a uniform standard for the above mentioned points as soon as possible in order to eliminate these controversial conclusions. Overall, in this experiment, our findings point to a negative correlation between the left middle frontal gyrus ALFF and SAS and VAS in LDHCP patients, while the right superior temporal gyrus was positively correlated with SAS and VAS, and the left dorsolateral superior frontal gyrus and right middle frontal gyrus were negatively correlated with VAS and SAS, respectively, which is in accordance with the results of previous relevant studies. There are also limitations to our study. First, we did not differentiate further subgroups of lumbar disc herniation in terms of the degree and direction of herniation. Secondly, many patients were unable to provide the specific time of the first episode of LDHCP, so we did not collect LDHCP duration as a factor in this study. Third, the pain-focused position was not specifically limited in this study. These factors should be progressively modified in future studies, taking into account the actual clinical situation.

## Conclusion

This study describes the regions of altered spontaneous neural activity in LDHCP patients compared to HCs. The right superior temporal gyrus, dorsolateral superior frontal gyrus and middle frontal gyrus may have important roles in regulating negative emotions and pain, providing new evidence to support the exploration of pathological mechanisms in LDHCP.

## Data availability statement

The raw data supporting the conclusions of this article will be made available by the authors, without undue reservation.

## Ethics statement

The studies involving human participants were reviewed and approved by the Medical Research Ethics Committee of Yueyang Hospital of Integrated Traditonal Chinese and Western Medicine (approval number: 2021-67), Shanghai University of Traditonal Chinese Medicine, Shanghai, China. The patients/participants provided their written informed consent to participate in this study.

## Author contributions

MF and LK: designing research studies. CY, BZ, ZW, and SF: conduction of the study and data acquisition. CT, WH, and GG: analyzing data. XP and XL: collecting and organizing research literature. CT and GG: writing the manuscript. All authors discussed the results, commented on the manuscript, and carefully reviewed and approved the submitted version.

## Funding

This work was supported by the National Natural Science Foundation of China (grant numbers: 82030121, 82004493, 82105042, and 82205304); and Innovation Team and Talents Cultivation Program of National Administration of Traditional Chinese Medicine (ZYYCXTD-C-202008).

## Conflict of interest

The authors declare that the research was conducted in the absence of any commercial or financial relationships that could be construed as a potential conflict of interest.

## Publisher’s note

All claims expressed in this article are solely those of the authors and do not necessarily represent those of their affiliated organizations, or those of the publisher, the editors and the reviewers. Any product that may be evaluated in this article, or claim that may be made by its manufacturer, is not guaranteed or endorsed by the publisher.

## Footnotes

^1^ http://www.restfmri.net

^2^ http://www.fil.ion.ucl.ac.uk/spm
